# Supplementary material for: Circular RNA circTmem241 drives group III innate lymphoid cell differentiation via initiation of Elk3 transcription
Source: Nat Commun. 2022 Aug 11;13:4711. doi: 10.1038/s41467-022-32322-z (PMC9372085; doi:10.1038/s41467-022-32322-z)
Supplement: Supplementary file 1 — Supplementary Information [file 41467_2022_32322_MOESM1_ESM.pdf]

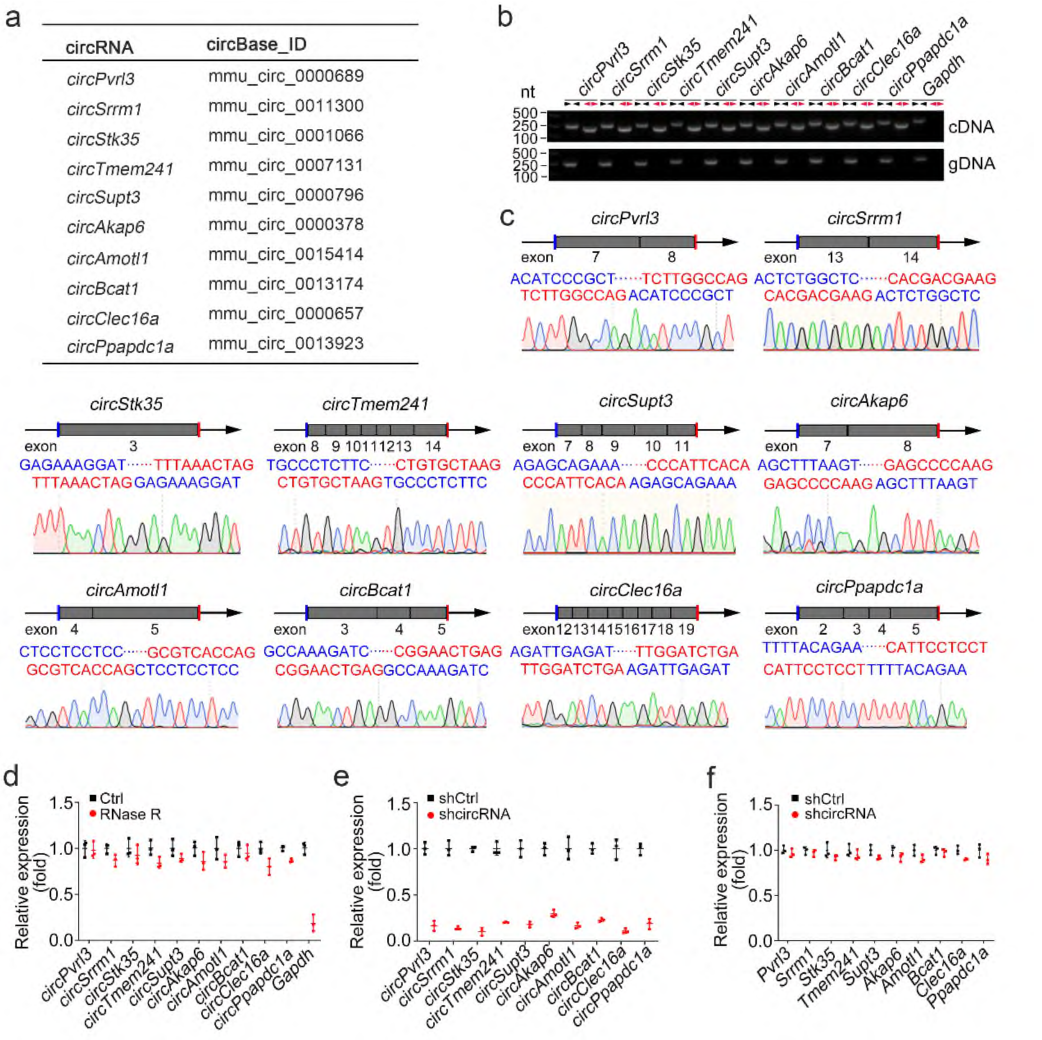

Supplementary Fig. 1. Validation of top 10 highly expressed circRNAs in ILC3s. (**a**) Information of top 10 highly expressed circRNAs. (**b**) Both convergent (black arrowheads) and divergent (red arrowheads) primers were used to amplify circRNAs from complementary DNA (cDNA) or genomic DNA (gDNA) in ILC3s. (**c**) CircRNAs were amplified by divergent primers and subjected to Sanger sequencing for validation of the back-splicing junction sites. (**d**) Total RNAs were extracted from ILC3s and treated with or without 3 U/μg RNase R at 37°C for 1 h, followed by RNA purification and qRT-PCR analysis of circRNAs. n=3 for each group. (**e, f**) After infected with virus expressing shRNAs or scramble vector, CHILPs (Lin^{-}CD25^{-}CD127^{+}Flt3$^{-}\alpha4\beta7^{+}$) were isolated for knockdown validation of circRNAs (**e**) and their parental genes (**f**) via qRT-PCR analysis. n=3 for each group. Data were analyzed by an unpaired Student's *t*-test and shown as means ± SD. Data are representative of at least three independent experiments. Source data are provided as a Source Data file.

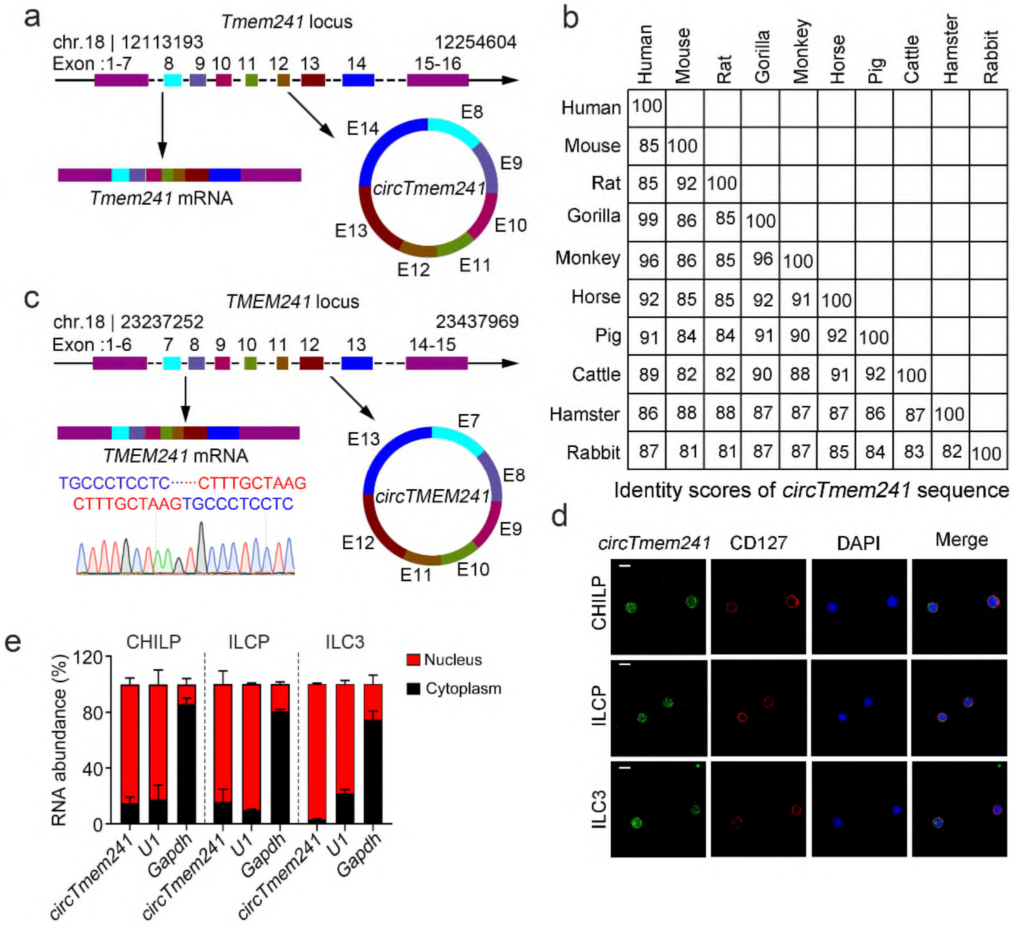

Supplementary Fig. 2. Analysis of *circTmem241* conservation across species. (**a**) Schematic representation of mouse *circTmem241*. (**b**) Conservation analysis of *circTmem241* in indicated vertebrate animals. (**c**) Validation of human *circTMEM241* in isolated human LPLs. Back-splicing site of *circTMEM241* was amplified by PCR, followed by Sanger sequencing. (**d**) FISH and IF staining of isolated CHILPs, ILCPs and ILC3s with indicated probes and antibodies. Scale bars, 10 μm (**e**) CHILPs (left), ILCPs (middle) and ILC3s (right) were sorted by FACS and subjected to nuclear and cytoplasmic isolation, followed by RNA extraction and qRT-PCR analysis of *circTmem241*. n=3 for each group. * *P* < 0.05, ** *P* < 0.01 and *** *P* < 0.001. Data were analyzed by an unpaired Student's *t*-test and shown as means ± SD. Data are representative of at least three independent experiments. Source data are provided as a Source Data file.

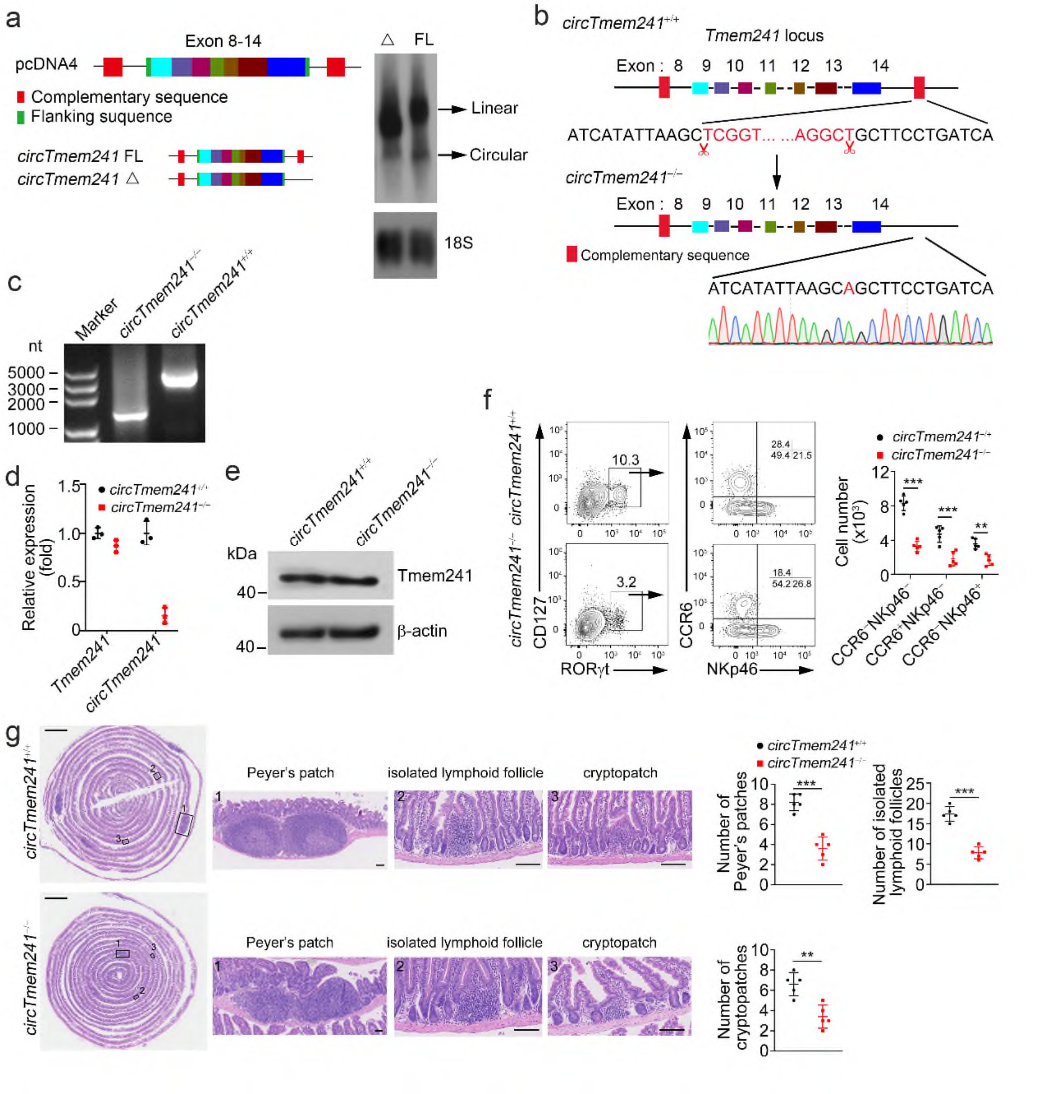

Supplementary Fig. 3. Generation of *circTmem241* knockout mice. (**a**) Diagram of minigene assay. Exons and complementary introns for *circTmem241* formation were cloned into pcDNA4 vector, followed by transfection into 293T cells. Total RNAs were extracted and *circTmem241* formation was measured by Northern blotting. (**b**) Construction diagram of *circTmem241* knockout mouse and DNA sequencing for knockout validation. (**c**) Genotyping presentation of *circTmem241* knockout mice by PCR analysis. (**d**) *Tmem241* and *circTmem241* gene expression in indicated ILC3s was tested via qRT-PCR analysis. n=3 for each group. (**e**) Tmem241 protein levels in ILC3s were detected by Western blotting. (**f**) Subsets of ILC3s ($CD3^{-}CD19^{-}CD45^{low}$ gated) were analyzed in small intestines of *circTmem241*$^{+/+}$ and *circTmem241*$^{-/-}$ mice by FACS. n=5 for each group. (**g**) Small intestines from *circTmem241*$^{+/+}$ and *circTmem241*$^{-/-}$ mice were collected and subjected to HE staining, followed by analysis of indicated gut-associated lymphoid tissues. n=5 for each group. Data were analyzed by an unpaired Student's *t*-test and shown as means ± SD. Data are representative of at least three independent experiments. Source data are provided as a Source Data file.

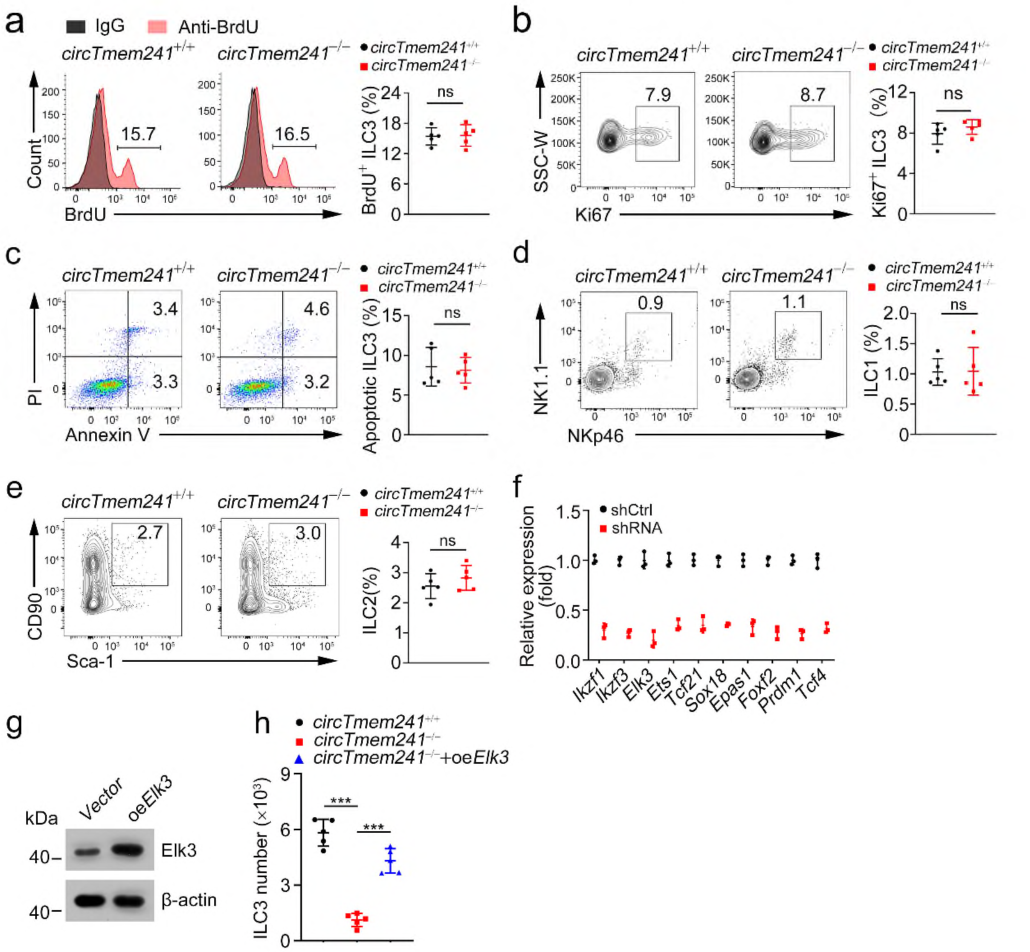

Supplementary Fig. 4. *CircTmem241* deficiency does not affect proliferation or cell death of ILC3s. (**a**) 100mg/kg dosage of BrdU was intraperitoneally injected into *circTmem241*$^{+/+}$ and *circTmem241*$^{-/-}$ mice and BrdU^{+} ILC3s were analyzed 18 h later by FACS. n = 5 for each group. (**b**) Proliferation analysis of Ki67^{+} ILC3s in *circTmem241*$^{+/+}$ and *circTmem241*$^{-/-}$ mice by FACS. n = 5 for each group. (**c**) Apoptosis detection of ILC3s by PI plus Annexin-V staining. n = 5 for each group. (**d, e**) FACS analysis of ILC1s and ILC2s. Percentages were counted in the right panel. n = 5 for each group. CHILPs were isolated for *in vitro* differentiation assay. (**f**) Top 10 downregulated TFs in ILCPs were knocked down by shRNAs, knockdown efficiency was detected by qRT-PCR analysis. n=3 for each group. (**g**) Elk3 overexpression was validated by Western blotting. (**h**) Hematopoietic progenitor cells from *circTmem241*$^{+/+}$ and *circTmem241*$^{-/-}$ mice were infected with *Elk3*-expressing or scramble sequence and subjected to in vitro differentiation assay, followed by FACS analysis of ILC3s. n=5 for each group. *** $P < 0.001$. Data are shown as means ± SD. Data are representative of at least three independent experiments. Data were analyzed by an unpaired Student's *t*-test and shown as means ± SD. Data are representative of at least three independent experiments. Source data are provided as a Source Data file.

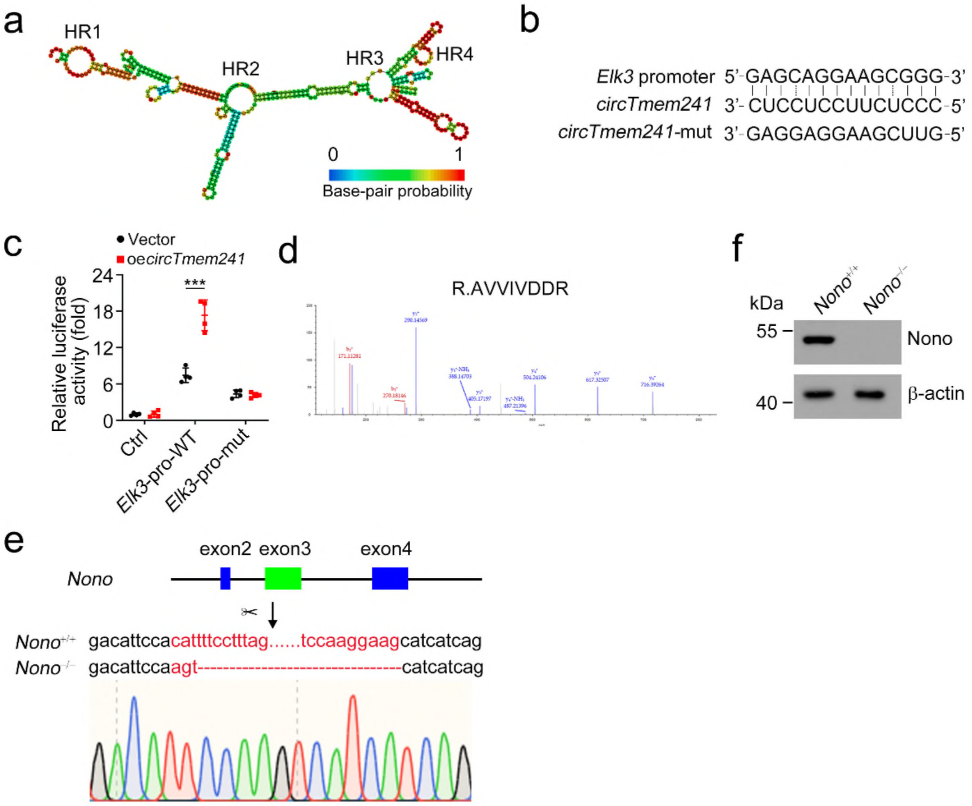

Supplementary Fig. 5. *circTmem241* interacts with Nono. (**a**) Secondary structure prediction of *circTmem241* by RNAfold Server (http://rna.tbi.univie.ac.at/) using minimum free energy (MFE) and partition function methods. 4 major hairpin regions (HRs) were identified. (**b**) Sequences of binding region between *circTmem241* transcript and *Elk3* promoter. Pairing region of *circTmem241* was mutated as indicated. (**c**) Luciferase reporter assay was performed using indicated *Elk3* promoters to validate *circTmem241* function on *Elk3* transcription activation. WT, wild-type; mut, mutation. n=4 for each group. (**d**) Peptides of Nono were identified by mass spectrometry. (**e**) Diagram of *Nono* knockout by *in vitro* AAV delivery of targeting guide RNAs into hematopoietic progenitor cells from *Vav-Cre;Cas9-KI* mice, which was validated by DNA sequencing. (**f**) Knockout of Nono was detected by Western blotting. Data were analyzed by an unpaired Student's *t*-test and shown as means ± SD. Data are representative of at least three independent experiments. Source data are provided as a Source Data file.

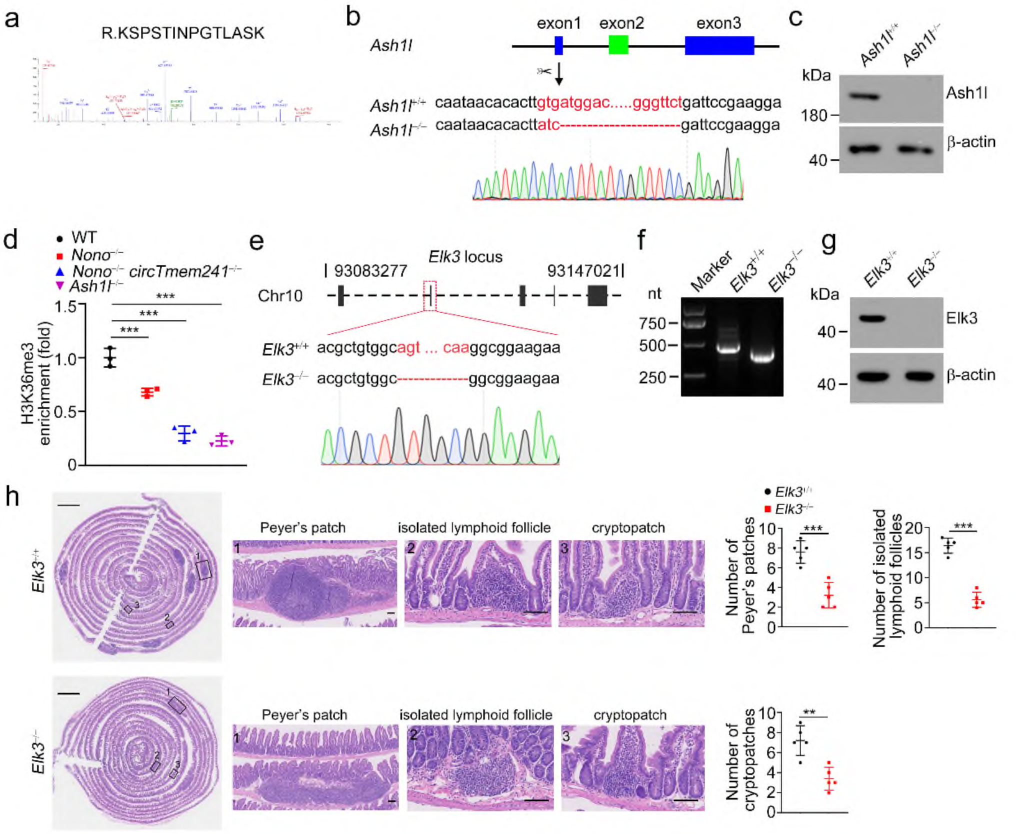

Supplementary Fig. 6. Generation of Ash1l deleted hematopoietic progenitor cells and *Elk3* knockout mice. (**a**) Peptides of Ash1l were identified by mass spectrometry. (**b**) Diagram of *Ash1l* knockout by *in vitro* AAV delivery of targeting guide RNAs into hematopoietic progenitor cells from *Vav-Cre;Cas9-KI* mice, which was validated by DNA sequencing. (**c**) Knockout of Ash1l was detected by western blotting. (**d**) ChIP assay was conducted using indicated hematopoietic progenitor cells to measure H3K36me3 enrichment on *Elk3* promoter. n=3 for each group. (**e**) Diagram of *Elk3* knockout mice and validation by DNA sequencing. (**f**) Genotyping of *Elk3* knockout mice by PCR analysis. (**g**) Western blotting analysis of *Elk3* expression in *Elk3*$^{+/+}$ and *Elk3*$^{-/-}$ mice. (**h**) Small intestines from *Elk3*$^{+/+}$ and *Elk3*$^{-/-}$ mice were collected and subjected to HE staining, followed by analysis of indicated gut-associated lymphoid tissues. n=5 for each group. *** $P < 0.001$. Data were analyzed by an unpaired Student's *t*-test and shown as means ± SD. Data are representative of at least three independent experiments. Source data are provided as a Source Data file.

Supplementary Table 1. Sequences of wild type and mutant *circTmem241* in this study

Loop	Wild-type sequence	Mutant sequence
HR1	5’-CCUCUUCCUCCUCUGU-3’	5’-ACAGAGGAUCCUCUGU-3’
HR2	5’-CUUCUCAU-3’	5’-CUUCGAAG-3’
HR3	5’-GACAUGCU-3’	5’-GACAUAUGCU-3’
HR4	5’-AUGUCU-3’	5’-AUGUCGACAU-3’

Supplementary Table 2. sgRNAs for CRISPR/Cas9-mediated mouse construction and *in vitro* knockout in this study

Genes	sgRNA sequences
circTmem241 (up)	5’-AAGCATCATATTAAGCTCGGTGG-3’
circTmem241 (down)	5’-AAGTATGTGGGTCTGTAAGGAGG-3’
Elk3 (up)	5’-GGTCCAGCAGCAAGTGCAAGAGG-3’
Elk3 (down)	5’-CGAGTTCAAGCTCCTCAAGGCGG-3’
Nono (up)	5’-TTGTACCCTAAAGGAAAATGTGG-3’
Nono (down)	5’-TGATGCTGATGATGCTTCCTTGG-3’
Ash1l (up)	5’-TCACAAGTGTGTTATTGCCAAGG -3’
Ash1l (down)	5’-GGATTGGGTTCTGATTCCGAAGG-3’

Supplementary Table 3. qPCR primers used in this study

Genes	Forward	Reverse
circPvrl3	5'-AATTCCTTCTCTCCCTCAGAAA-3'	5'-CAAAGACGGTGATGATGAAGAG-3'
circSrrm1	5'-TAGTAGAAGGAGGAGAAGTCCC-3'	5'-GTTCTTTACATGGGATCTCTTGG-3'
circStk35	5'-TGATGCTTTTGAACTTGAAACC-3'	5'-CTCCATGACAAACCAGAGATAG-3'
circTmem241	5'-GAGTTTTGGGATTCTTCCTCAT-3'	5'-GATCAAACTGAGAGTCTTGGAA-3'
circSupt3	5'-CCTCTCTCCCCATTCACAAGAG-3'	5'-AAACTTAACTGTCGGCTTTCAC-3'
circAkap6	5'-GAAGGACATGCTGAAGATGATT-3'	5'-GTGACTGTCTACGTTCAACTTAA-3'
circAmotl1	5'-CCATGAGATGGTCAAACCTTAC-3'	5'-CTTCATGCCTCCCAGAATAGAG-3'
circBcat1	5'-CCTAAAGTGTATTCTTCAGCTTCTA-3'	5'-GTCAGTAAACGTAGCTCCAAAG-3'
circClec16a	5'-GGTATTCTTCATGCTGCGTTCC-3'	5'-CTTCTCCTCGTCTGTGGTGTTC-3'
circPpapdc1a	5'-GATTTCTTTTACCGCTGCTTTC-3'	5'-TTATAGAGCCAGATCTCTTCCG-3'
Pvrl3	5'-GGTCTTCAGCCCTCTAATGGA-3'	5'-GGGTTGCGTGGGTGATGTT-3'
Srrm1	5'-CCAAGACGACGACCATCTCC-3'	5'-GACGAGCTACTTCCAGACAGG-3'
Stk35	5'-AGCTACGGCGTGGTTTATGAG-3'	5'-AATTCTGCTAGTGCCAACTCC-3'
Tmem241	5'-CCAGAATGGCTGACCATCAAG-3'	5'-ATCCCCAGAACCCACGGAG-3'
Supt3	5'-AAAGGCATTGACGAGGATGAC-3'	5'-CCTGTCTGGTCGATAGAGTTGAT-3'
Akap6	5'-AGGACTCATCTCAAGTGATCCTT-3'	5'-TTCAGCAGCGTTTGTCAGTGT-3'
Amotl1	5'-ATGTAGCCTCTGGAAGAGTGT-3'	5'-TGAGTGAGGGTTTCCGTGGA-3'
Bcat1	5'-CCCATCGTACCTCTTTCACCC-3'	5'-GGGAGCGTGGGAATACGTG-3'
Clec16a	5'-GCAAGTCCTCCCGCAACAT-3'	5'-GAGCAGGTTCCGATTTTGTTCT-3'
Ppapdc1a	5'-CCCTTCTCTTCGGGGTCTTTG-3'	5'-CGGGTAGGTATGTTGTCTGACT-3'
Ikzf1	5'-ATGTCCCAAGTTTCAGGAAAG-3'	5'-GCACGCCCATTCTCTTCATC-3'
Ikzf3	5'-CTGAATGACTACAGCTTGCCC-3'	5'-GCTCCGGCTTCATAATGTTCT-3'
Elk3	5'-TCCTCACGCGGTAGAGATCAG-3'	5'-GTGGAGGTACTCGTTGCGG-3'
Ets1	5'-CCTATCAGCTCGGAAGAACTC-3'	5'-TCTTGCTTGATGGCAAAGTAG-3'
Tcf21	5'-CCCACTAAGAAAAGCCCGCTC-3'	5'-CCGTTCTCGTACTTGTCGTTG-3'
Sox18	5'-CCTGTCACCAACGTCTCGC-3'	5'-GCAACTCGTCGGCAGTTTG-3'
Epas1	5'-CTGAGGAAGGAGAAATCCCGT-3'	5'-TGTGTCCGAAGGAAGCTGATG-3'
Foxf2	5'-CGTCCTCTTCTAACTCCGTC-3'	5'-TGTACGAGTAAGGAGGCTTCT-3'
Prdm1	5'-TTCTCTTGGAAAAACGTGTG-3'	5'-GGAGCCGGAGCTAGACTTG-3'
Tcf4	5'-AAGTCCGAAAAGTTCCTCCG-3'	5'-CTCCATAGCCCGGCTGATT-3'
Gapdh	5'-AGGTCGGTGTGAACGGATTTG-3'	5'-TGTAGACCATGTAGTTGAGGTCA-3'
U1	5'-CGCTTACAAGCACGCAGATG-3'	5'-CCCGAATGCCTGATATTCACAT-3'
18S	5'-TAACGAACGAGACTCTGGCAT-3'	5'-CGGACATCTAAGGGCATCACAG-3'

Supplementary Table 4. Primers for PCR amplification of top 10 circRNAs (Con: convergent; Div: divergent primer)

Genes	Forward	Reverse
circPvrl3 (Con)	5’-CATCCCGCTTACGCAGACCT-3’	5’-TGGCCAAGAAGGTCTTTCTG-3’
circPvrl3 (Div)	5’-CTTGACAAAGTAATCGACCTTC-3’	5’-GTCAGCAACACAGTCACAAAG-3’
circSrrm1 (Con)	5’-GCTCCTCTTCCACCTCAGAAG-3’	5’-GCTGGAGAAGGAGACCTTCGC-3’
circSrrm1 (Div)	5’-GCCAGAAGGCGAAGGTCTCCT-3’	5’-GCACTCCGAGAAGGGGAATGTC-3’
circStk35 (Con)	5’-TGTCCTGTCCCGGAGACCTG-3’	5’-GCCACAAGCTGAGGACAGCCAG-3’
circStk35 (Div)	5’-CACAGGACCGACCTGATGCT-3’	5’-CAGGTCTCCGGGACAGGACAT-3’
circTmem241 (Con)	5’-CTTGGCTGCTGCAGGGTGCCT-3’	5’-GGAACCTGTAGAAATACAAGA-3’
circTmem241 (Div)	5’-GTTTTGGGATTCTTCCTCATG-3’	5’-CGAAGTATTTTGTAAGAACCT-3’
circSupt3 (Con)	5’-CAAATTCCGAGACTGGCTGGA-3’	5’-GTGTGTTCTCAGGAGAGTCTG-3’
circSupt3 (Div)	5’-TGTGGTGTCGAGGCTCACAGC-3’	5’-GTCCAGCCAGTCTCGGAATTTG-3’
circAkap6 (Con)	5’-CTTTAAGTTGAACGTAGACAG-3’	5’-GTCCCCTCCTCGTCCTCCACAG-3’
circAkap6 (Div)	5’-GCAACACAGCTGGATTCTCAG-3’	5’-CATGTCCTTCAGTCCTGCTTTG-3’
circAmotl1 (Con)	5’-GCATGAAGCATCTACTTTGAC-3’	5’-CCCTGTGGCCTGGTGCTGAATG-3’
circAmotl1 (Div)	5’-AGATGGTCAAACCTTACCCAG-3’	5’-GTCAAAGTAGATGCTTCATGCCT-3’
circBcat1 (Con)	5’-GATCTCATCATCACACCAGCCA-3’	5’-GCACATTCTATCCATGTTGAGGT-3’
circBcat1 (Div)	5’-GCTCCTAAAGTGTATTCTTCAG-3’	5’-GAGGACCACTCCACCGTCAGC-3’
circClec16a (Con)	5’-GAGATGGTGATCATGAAGCTTG-3’	5’-CTGGGAGCTGAATTCGTTTTAG-3’
circClec16a (Div)	5’-TCCCTGTCACTGCAGCTGCG-3’	5’-CACTGTGCATTCTCTGAGTTC-3’
circPpapdc1a (Con)	5’-GGATCCATTCCAGAGAGTCAT-3’	5’-GCGAGGTCTTCCCACTATTAG-3’
circPpapdc1a (Div)	5’-TGCTTTCCAGATGGGGTGATG-3’	5’-GTAGGTATGTTGTCTGACTGC-3’
Gapdh (Con)	5’-GCGCCTGGTCACCAGGGCTG-3’	5’-GACTCCACGACATACTCAGC-3’
Gapdh (Div)	5’-GCTGAGTATGTCGTGGAGTC-3’	5’-GCAGCCCTGGTGACCAGGCG-3’

Supplementary Table 5. Primers for ChIP assay in this study

Genes	Forward	Reverse
Elk3 pro#1	5'-TATGGTAGAGAAAAGCTCGCTTAGG-3'	5'-GTGCCCGCCCCGCTTCCTGCT-3'
Elk3 pro#2	5'-TATAGTGCTGCTGTTAAATATGCC-3'	5'-GCATAGAAGAAGAGTTTAGGGACAG-3'
Elk3 pro#3	5'-GGAGTCCAGTCTTGAAACCACAG-3'	5'-TGGAGGCAGTTTTAGAGTTGG-3'
Elk3 pro#4	5'-CCATGTACTCTCATGCCAAACG-3'	5'-GCCCCTGAGGACAATGGCCAGGAT-3'
Elk3 pro#5	5'-GGCAAGGAAATGTCAGAATGTTG-3'	5'-GCTCATTAAAGCCCTAATTGTAAC-3'
Elk3 pro#6	5'-GTCCTCAGTGTATAACCAGCAGG-3'	5'-TGCTATCTCTCTGAAAGATTGC-3'
Elk3 pro#7	5'-GCTGAGTGACTGGAGAAGCCAACT-3'	5'-GTTATACACTGAGGACCAGGCTTG-3'
Elk3 pro#8	5'-GTTCTGCCTTTTCTGGGAGAGGTG-3'	5'-GCAAAACTCAGACTGTGTCCCT-3'
Elk3 pro#9	5'-GAACCTTTCTTGACACAACCTC-3'	5'-GCAGAACTATTGGCGCAGTGAG-3'
Elk3 pro#10	5'-GTGCTGGAGATTGAACTCATGG-3'	5'-GGTTCGTCATTTGTAAGTGGT-3'

Supplementary Table 6. shRNA sequences used in this study

Genes	shRNA sequences
circPvrl3	5’- TGGCCAGACATCCCGCTTA-3’
circSrrm1	5’- CGAAGACTCTGGCTCCTCT -3’
circStk35	5’- GTTTTTAAACTAGGAGAAA -3’
circTmem241	5’-TGCTAAGTGCCCTCTTCCT-3’
circSupt3	5’- TCACAAGAGCAGAAAGACA -3’
circAkap6	5’-GCCCCAAGAGCTTTAAGTT-3’
circAmotl1	5’-TCACCAGCTCCTCCTCCCA-3’
circBcat1	5’-TCGGAACTGAGGCCAAAGA-3’
circClec16a	5’-ATCTGAAGATTGAGATGGT-3’
circPpapdc1a	5’-GTGGGAAGTTTTACAGAAT-3’
Ikzf1	5’-GGCATGTACCCAGTCATTA-3’
Ikzf3	5’-GCGACAGAGATGAGAACA-3’
Elk3	5’-GCAGAGCGCTGAGATACTA-3’
Ets1	5’-GAGCCAGTCGTGGTAAACT-3’
Tcf21	5’-GTACGAGAACGGTTACATT-3’
Sox18	5’-GCGACCATCCCAACTACAA-3’
Epas1	5’-GACCAGCAAATGGATAACT-3’
Foxf2	5’-GCCTCGTCCTCTTCTAACT-3’
Prdm1	5’-GGAGGACGCTGATATGACT-3’
Tcf4	5’-GTCCACGTTCCATCGTAGT-3’
