# Supplementary material for: Circular RNA circTmem241 drives group III innate lymphoid cell differentiation via initiation of Elk3 transcription
Source: Nat Commun. 2022 Aug 11;13:4711. doi: 10.1038/s41467-022-32322-z (PMC9372085; doi:10.1038/s41467-022-32322-z)
Supplement: Supplementary file 3 — Reporting Summary [file 41467_2022_32322_MOESM3_ESM.pdf]

nature portfolio

Corresponding author(s): Zusen Fan

Last updated by author(s): Jun 20, 2022

Reporting Summary

Nature Portfolio wishes to improve the reproducibility of the work that we publish. This form provides structure for consistency and transparency in reporting. For further information on Nature Portfolio policies, see our Editorial Policies and the Editorial Policy Checklist.

Statistics

For all statistical analyses, confirm that the following items are present in the figure legend, table legend, main text, or Methods section.

n/a	Confirmed	
☐	☒	The exact sample size (*n*) for each experimental group/condition, given as a discrete number and unit of measurement
☐	☒	A statement on whether measurements were taken from distinct samples or whether the same sample was measured repeatedly
☐	☒	The statistical test(s) used AND whether they are one- or two-sided<br>*Only common tests should be described solely by name; describe more complex techniques in the Methods section.*
☒	☐	A description of all covariates tested
☐	☒	A description of any assumptions or corrections, such as tests of normality and adjustment for multiple comparisons
☐	☒	A full description of the statistical parameters including central tendency (e.g. means) or other basic estimates (e.g. regression coefficient) AND variation (e.g. standard deviation) or associated estimates of uncertainty (e.g. confidence intervals)
☐	☒	For null hypothesis testing, the test statistic (e.g. *F*, *t*, *r*) with confidence intervals, effect sizes, degrees of freedom and *P* value noted<br>*Give P values as exact values whenever suitable.*
☒	☐	For Bayesian analysis, information on the choice of priors and Markov chain Monte Carlo settings
☒	☐	For hierarchical and complex designs, identification of the appropriate level for tests and full reporting of outcomes
☒	☐	Estimates of effect sizes (e.g. Cohen's *d*, Pearson's *r*), indicating how they were calculated

Our web collection on statistics for biologists contains articles on many of the points above.

Software and code

Policy information about availability of computer code

Data collection	Flow cytometry data were collected using FACS Aria IIIu instruments;<br>qRT-PCR data were collected by QuanStudio 1 biosystems;<br>Confocal images were obtained by Nikon A1R+ microscopy.
Data analysis	Data were analyzed by R studio Version 1.3, Microsoft Excel Version 2016 and GraphPad Prism Version 8.3. All flow cytometry data were analyzed with FlowJo Version 10.0. Adobe Photoshop CS6 and Imaris Version 9.0 were used for figure presentation. One-tailed unpaired Student's t-test was performed using GraphPad Prism Version 8.3. No computer code was used.

For manuscripts utilizing custom algorithms or software that are central to the research but not yet described in published literature, software must be made available to editors and reviewers. We strongly encourage code deposition in a community repository (e.g. GitHub). See the Nature Portfolio guidelines for submitting code & software for further information.

Data

Policy information about availability of data

All manuscripts must include a data availability statement. This statement should provide the following information, where applicable:

- Accession codes, unique identifiers, or web links for publicly available datasets
- A description of any restrictions on data availability
- For clinical datasets or third party data, please ensure that the statement adheres to our policy

The source data for Figs. 1-7 and Supplementary Figs. 1-6 are provided as a Source Data file. RNA sequencing data were deposited to the GEO database (GSE201065). All other data are included in the supplementary information and also available from the authors upon reasonable requests.

Field-specific reporting

Please select the one below that is the best fit for your research. If you are not sure, read the appropriate sections before making your selection.

- [x] Life sciences
- [ ] Behavioural & social sciences
- [ ] Ecological, evolutionary & environmental sciences

For a reference copy of the document with all sections, see nature.com/documents/nr-reporting-summary-flat.pdf

Life sciences study design

All studies must disclose on these points even when the disclosure is negative.

Sample size	No statistical methods were applied to predetermine sample size. We chose the sample size based on previous studies that showed significant results. Where statistical analysis was applied in this study, at least three independent repeated experiments were performed which is similar to those in previous publications.
Data exclusions	No data were excluded from the anaylsis.
Replication	All experiments were prepared with at least three biological repeats and all attempts at replication were successful.
Randomization	For imaging and mice experiments, each cell or mouse was randomly chosen. For other experiments randomization is not relevant, cells in bulk were used for western blot, qPCR or FACS etc.
Blinding	Data collection and analysis were not conducted blind to the experimental conditions. There is no special blinding as the analysis were performed by machine or software where blinding is not necessary to reduce bias.

Reporting for specific materials, systems and methods

We require information from authors about some types of materials, experimental systems and methods used in many studies. Here, indicate whether each material, system or method listed is relevant to your study. If you are not sure if a list item applies to your research, read the appropriate section before selecting a response.

Materials & experimental systems

n/a	Involved in the study	
[ ]	[x]	Antibodies
[ ]	[x]	Eukaryotic cell lines
[x]	[ ]	Palaeontology and archaeology
[ ]	[x]	Animals and other organisms
[x]	[ ]	Human research participants
[x]	[ ]	Clinical data
[x]	[ ]	Dual use research of concern

Methods

n/a	Involved in the study	
[x]	[ ]	ChIP-seq
[ ]	[x]	Flow cytometry
[x]	[ ]	MRI-based neuroimaging

Antibodies

Antibodies used	For Immunofluorescence: Anti-Nono (Proteintech, #11058-1-AP, 1:500), anti-RORγt (eBioscience, #14-6988-80, AFKJS-9, 1:100), FITC anti-CD3 (eBioscience, # 11-0031-82,145-2C11, 1:300) and anti-CD127/IL-7R (Proteintech, Cat# 17626-1-AP, 1:200). Secondary antibodies: Alexa Flour 594 donkey anti-rat (#A-21209, 1:500), Alexa Flour 647 goat anti-rabbit (#A-21244, 1:500) and Alexa Flour 594 donkey anti-mouse IgG (#A-21203, 1:500) were purchased from ThermoFisher (Waltham, USA).<br>For ChIP experiments: Anti-H3K4me3 (Cell Signalling Technology, #9751S, C42D8, 2microgram/sample), anti-H3K36me3 (Bioworld, #BS7239, 2microgram/sample) and anti-Ash1l (Bioss, #bs-9671R, 2 microgram/sample).<br>For western blot: Anti-Elk3 (Novus Biologicals, #NBP1-83960, 1:500), anti-Tmem241 (Sinobiological, #203644-T32, 1:400), anti-Ash1l (Bioss, #bs-9671R, 1:500), anti-Nono (Proteintech, #66361-1-Ig, 2A2B10, 1:200), anti-Flag tag antibody (Abcam, #ab205606, 1:1000), anti-β-actin (Beijing Ray Antibody Biotech, #RM2001, 1:1000), HRP goat anti-mouse IgG (Beijing Ray Antibody Biotech, #RM3001, 1:3000) and HRP goat anti-rabbit IgG (Beijing Ray Antibody Biotech, #RM3002, 1:3000).<br>For FACS experiments: Anti-Lineage cocktail (#88-7772-72, 1:300), Anti-CD127 (#46-1273-82, A7R34, 1:200), anti-Sca-1 (#11-5981-85, D7, 1:200), anti-Flt3 (#12-1351-82, A2F10, 1:300), anti-α4β7 (#17-5887-82, DATK32, 1:200), anti-Id2 (#12-9475-80, ILCID2, 1:300), anti-PLZF (#12-9320-80, Mags.21F7, 1:200), anti-Eomes (#53-4875-82, Dan11mag, 1:200), anti-NKp46 (#11-3351-80, 29A1.4, 1:200), anti-NK1.1 (#17-5941-82, PK136, 1:300), anti-CD45.1 (#45-0453-80, A20, 1:500), anti-CD45.2 (#12-0454-82, 30-F11, 1:500), anti-CD25 (#12-0251-81, PC61.5, 1:300), anti-Gata3 (#25-9966-41, TWAJ, 1:200), anti-RORγt (#17-6981-80, AFKJS-9, 1:200), anti-CD19 (#12-0193-81, 1D3, 1:500), anti-KLRG1 (#17-5893-82, 2F1, 1:200), anti-CD90 (#11-0903-81, HIS51, 1:500), anti-IL-22 (# 17-7222-80, IL22JOP, 1:200), anti-Ki67 (#11-5698-80, SolA15, 1:300), anti-BrdU (#11-5071-41, BU20A, 1:200) and anti-PD1 (#25-9985-80, J43, 1:300) were purchased from eBiosciences (San Diego, USA). Anti-Bcl11b/Ctip2 (#ab240636, EPR23120-25, 1:200) was from Abcam. Anti-c-Kit (#105811, 2B8, 1:300), anti-CD3 (#100206, 17A2, 1:500), anti-CD150 (#115915, TC15-12F12.2, 1:200), anti-CD48 (#103417, HM48-1, 1:300) and anti-CD49a (#1426003, HMα1, 1:200) were purchased from Biolegend (California, USA).

Validation

Antibodies for Immunofluorescence were validated on control mice intestinal frozen sections or FACS isolated ILCPs before using for experiments. Anti-Nono (Proteintech, #11058-1-AP, 1:500) antibody was used by previous research: Brieño, Moak et al. Characterization of telomeric repeat-containing RNA (TERRA) localization and protein interactions in primordial germ cells of the mouse. Biol Reprod. 2019 Apr 1;100(4):950-962. doi: 10.1093/biolre/ioy243. Other antibodies were validated by previous work of our lab: Liu, B et al. Long noncoding RNA lncKdm2b is required for ILC3 maintenance by initiation of Zfp292 expression. Nat Immunol 18, 499–508 (2017). https://doi.org/10.1038/ni.3712.
Antibodies for ChIP experiments were validated by cell lysates of ILCPs from control mice prior to use for experiments. Anti-H3K4me3 (Cell Signalling Technology, #9751S, C42D8, 2microgram/sample) antibody was validated by more than 300 citations: Liu C et al. CHD7 regulates bone-fat balance by suppressing PPAR-γ signaling. Nat Commun. 2022 Apr 13;13(1):1989. doi: 10.1038/s41467-022-29633-6. Anti-H3K36me3 (Bioworld, #BS7239, 2microgram/sample) and anti-Ash1l (Bioss, #bs-9671R, 2 microgram/sample) antibodies was validated using ChIP and western blot experiments by manufacturers and in our lab.
Antibodies for FACS experiments were validated by using bone marrow cells or lamina propria lymphocytes isolated from control mice before using for experiments. All these antibodies were used before by our lab: Liu B et al. An inducible circular RNA circKcnt2 inhibits ILC3 activation to facilitate colitis resolution. Nat Commun 11, 4076 (2020). https://doi.org/10.1038/s41467-020-17944-5.

Eukaryotic cell lines

Policy information about cell lines

Cell line source(s)

HEK293T cell line was obtained from ATCC. OP9-DL1 cell line was a gift from Liu lab (Academy of Military Medical Sciences).

Authentication

None of the cell lines were authenicated.

Mycoplasma contamination

HEK293T and OP9-DL1 cell lines were tested negative for mycoplasma contamination.

Commonly misidentified lines
(See ICLAC register)

None used

Animals and other organisms

Policy information about studies involving animals; ARRIVE guidelines recommended for reporting animal research

Laboratory animals

C57BL/6 mice were purchased from Charles River Laboratories. circTmem241-/-, Elk3-/- mice were generated by Tian lab from Key Laboratory of RNA Biology department. PLZF-GFPcre, Vav-Cre and Cas9-KI mice were purchased from the Jackson Laboratory. Sex-matched littermates were used for all experiments. Animals were housed under specific pathogen-free environment, with 12/12h dark/light cycles, 23±2°C and 40% humidity conditions. Experiments were performed in accordance with protocols approved by the Institutional Committee of Institute of Biophysics, Chinese Academy of Sciences. Mice of eight to ten-weeks of age were used for analysis unless described otherwise. Female and male mice were used.

Wild animals

No wild animals were used in this study.

Field-collected samples

This study did not involve sample collected from the field.

Ethics oversight

All animal experiments were approved by the Institutional Animal Care and Use Committee of Institute of Biophysics, Chinese Academy of Sciences. All animals were maintained under specific pathogen-free conditions at the Institute of Biophysics.

Note that full information on the approval of the study protocol must also be provided in the manuscript.

Flow Cytometry

Plots

Confirm that:

- [x] The axis labels state the marker and fluorochrome used (e.g. CD4-FITC).
- [x] The axis scales are clearly visible. Include numbers along axes only for bottom left plot of group (a 'group' is an analysis of identical markers).
- [x] All plots are contour plots with outliers or pseudocolor plots.
- [x] A numerical value for number of cells or percentage (with statistics) is provided.

Methodology

Sample preparation

The itestine were isolated and attaching mesenteric lymph nodes (mLNs) were removed. Then the intestine was cut open longitudinally, followed by wash using phosphate buffer saline (PBS) five times. Afterwards, the intestine was cut into pieces and washed using solution I buffer (10 mM HEPES and 5mM EDTA in HBSS) five times, followed by digestion using solution II buffer (DNaseI, 5% FBS, 0.5 mg/ml collagenase II and collagenase III) three times. Finally, LPLs were filtered through 70 um strainers and utilized for FACS analyses

Instrument

FACSAria III instrument (BD Biosciences)

Software

Flowjo Version 10.0

Cell population abundance

About 1~2x10^5 cells were recorded by the cytometer for each sample.

Gating strategy

For flow cytometric analysis, HSC (Lin-Sca-1+c-Kit+CD150+CD48-), MPP (Lin-Sca-1+c-Kit+CD150-CD48+), CLP (Lin-CD127+c-Kit intSca-1intFlt3+α4β7-), α4β7+CLP (Lin-CD127+c-KitintSca-1intFlt3+α4β7+), CHILP (Lin-CD25-CD127+Flt3-α4β7+Id2+), ILCP (Lin-CD127+Flt3-c-Kit+α4β7+PLZFGFP), ILC1P (Lin-CD127+Eomes-CD49a+NK1.1+NKp46+), ILC2P (Lin-CD127+CD45+Flt3-CD117-Sca-1+CD25+Gata3+), ILC3P (Lin-CD25-CD127+α4β7intGata3loBcl11b+Id2+RORγt+), siILC1 (CD3-CD19-CD127+CD45+NK1.1+NKp46+), siILC2 (CD3-CD19-CD127+CD45+KLRG1+Sca-1+), siILC3 (Lin-CD127+CD45+RORγt+), NK1.1+ NK, CD19+ B and CD3+ T populations were analyzed or sorted with a FACSAria III instrument (BD Biosciences). PLZFGFPcre mice were used for ILCP (Lin-CD127+α4β7+PLZFGFP) isolation and Id2+/GFP mice were used for CHILP (Lin-CD25+CD127+Flt3+α4β7+Id2GFP) isolation by FACS.

☒ Tick this box to confirm that a figure exemplifying the gating strategy is provided in the Supplementary Information.
